# Glutathione in the Nervous System as a Potential Therapeutic Target to Control the Development and Progression of Amyotrophic Lateral Sclerosis

**DOI:** 10.3390/antiox10071011

**Published:** 2021-06-23

**Authors:** Kiyoung Kim

**Affiliations:** 1 Department of Medical Biotechnology, Soonchunhyang University, Asan 31538, Korea; kiyoung2@sch.ac.kr; Tel.: +82-41-413-5024; Fax: +82-41-413-5006; 2 Department of Medical Sciences, Soonchunhyang University, Asan 31538, Korea

**Keywords:** glutathione, oxidative stress, amyotrophic lateral sclerosis, neurogenerative disease

## Abstract

Amyotrophic lateral sclerosis (ALS) is a rare neurological disorder that affects the motor neurons responsible for regulating muscle movement. However, the molecular pathogenic mechanisms of ALS remain poorly understood. A deficiency in the antioxidant tripeptide glutathione (GSH) in the nervous system appears to be involved in several neurodegenerative diseases characterized by the loss of neuronal cells. Impaired antioxidant defense systems, and the accumulation of oxidative damage due to increased dysfunction in GSH homeostasis are known to be involved in the development and progression of ALS. Aberrant GSH metabolism and redox status following oxidative damage are also associated with various cellular organelles, including the mitochondria and nucleus, and are crucial factors in neuronal toxicity induced by ALS. In this review, we provide an overview of the implications of imbalanced GSH homeostasis and its molecular characteristics in various experimental models of ALS.

## 1. Introduction

Amyotrophic lateral sclerosis (ALS), also known as Lou Gehrig’s disease, is a progressive neurodegenerative disease characterized by the selective loss of motor neurons in the spinal cord and motor cortex [1,2]. Muscle weakness and degradation at 2–5 years after the onset of symptoms can result in fatal muscle dystrophy, paralysis, and death [3,4]. Most ALS cases (90–95%) occur sporadically with no clearly related risk factors, while approximately 5–10% of ALS cases are considered to be hereditary, attributed to various mutations in specific genes [1]. Pathogenic mutations in genes encoding *Cu/Zn-superoxide dismutase 1* (*SOD1*), *chromosome 9 open reading frame 72* (*C9orf72*), *optineurin* (*OPTN*), *p97/valosin-containing protein* (*VCP*), *TAR DNA-binding protein* (*TDP-43*), *fused in sarcoma* (*FUS*), *Ewing sarcoma* *breakpoint region 1* (*EWSR1*), and *TATA box-binding protein-associated factor 15* (*TAF15*), have been linked to the sporadic and familial forms of ALS [5,6,7,8,9]. There are only two FDA-approved drugs for ALS, and both extend patient lifespan by a few months [10,11]. Therefore, there is an urgent demand for the development of therapeutics for ALS.

*SOD1*, the first discovered ALS-linked gene, codes for an important enzyme in the defense mechanism against oxidative stress. Mutations in the *SOD1* gene account for approximately 20% of familial ALS cases [12]. Previous studies have suggested that mutant SOD1 forms insoluble aggregates in the mitochondria and induces mitochondrial defects that lead to cell death [13]. Recently, dominant mutations in specific genes coding for different RNA-binding proteins with prion-like domains, including *TDP-43*, *FUS*, *EWSR1*, and *TAF15*, have been found in sporadic and familial ALS cases [14,15,16,17]. Several studies have discovered mutations and functions of RNA-binding proteins in the pathogenesis of ALS. The expression of wild-type or mutant human TDP-43 in mice has been found to result in the degeneration of motor neurons [18]. There is evidence of ALS onset as a result of the presence of cytoplasmic inclusions in TDP-43 mutants in familial and sporadic ALS patients [3]. Furthermore, FUS is involved in RNA metabolism, including RNA processing, splicing, transport, and translation, by regulating cellular localization and degradation [9,15]. FUS is also localized in the nucleus and has been identified as a component of cytoplasmic inclusions in patients with familial ALS [19]. ALS-linked mutations in FUS lead to the mislocalization of FUS to the cytoplasm from the nucleus [20,21]. A recent study found a missense mutation in the *EWSR1* gene in patients with ALS [17]. Similar to other FET proteins, the mutant EWSR1 protein is mislocalized in the cytoplasm of the spinal cord [22]. The overexpression of EWSR1, namely EWSR1^G511A^ and EWSR1^P552L^, in neurons leads to neuronal dysfunction in *Drosophila* [17]. In addition, mutations in the *TAF15* gene have been implicated in the pathogenesis of familial and sporadic ALS, and the TAF15 protein also forms prion-like aggregates in the cytoplasm of spinal cord neurons of rats, leading to increased neuronal toxicity [16,23,24]. The property of cytoplasmic aggregate formation in several ALS-linked proteins has been suggested to play a critical role in the development and progression of ALS. Therefore, the clearance and degradation of cytoplasmic aggregates in motor neurons could be a therapeutic strategy for ALS. However, despite efforts by several research groups, the pathogenic mechanisms underlying neurodegeneration through gene mutations and the screening of potential therapeutics remain poorly understood.

Oxidative stress arises from an imbalance between the production of reactive oxygen species (ROS) and the antioxidant system, which removes oxidative damage in cells and neurons. Therefore, maintaining this balance is critical in the pathogenesis of neurodegenerative diseases, including ALS. Numerous pathological studies have found evidence of increased oxidative stress in ALS. Previous studies have revealed increased oxidative damage to proteins in the tissue of ALS patients postmortem. Increased levels of protein carbonyl were observed in the spinal cord and motor cortex of patients with sporadic ALS [25]. Furthermore, several biomarkers for antioxidant defense and ROS damage are altered in the peripheral tissues and cerebrospinal fluid of patients with ALS [26,27]. The activity and expression of SOD, catalase, glutathione reductase (GR), and glutathione transferase (GST) are also reduced in the cerebrospinal fluid or peripheral blood mononuclear cells of patients with familial or sporadic ALS [28,29,30]. The reduced form of glutathione (GSH), a tripeptide, is well-known to non-enzymatically react with ROS to scavenge free radicals. A reduction in the GSH/oxidized glutathione (GSSG) ratio and GSH levels in cerebrospinal fluid has also been observed in patients with ALS [31]. Thus, the antioxidant defense mechanism against oxidative stress, which includes reactive species scavengers, is an important system in ALS, and the dysregulation of GSH homeostasis is believed to contribute to the development and progression of ALS. Although uncertainties remain regarding the mechanisms underlying the association between GSH and the dysfunction of neuronal cells and the specific functions of GSH that are critical for inducing neuronal toxicity, such as ROS production and protein aggregation in ALS pathogenesis, dysfunctional GSH metabolism, GSH-related enzymatic systems, such as GR and GST, and an imbalanced redox status are increasingly postulated to be crucial to the development and progression of ALS.

In this review, we provide an update on recent advances in our understanding of the relationship between GSH and the progression of ALS and an overview of the pathophysiological role of GSH deficiency in the brain. Specifically, we focus on evidence of aberrant GSH metabolism in various ALS models and summarize experimental studies supporting GSH redox imbalance as the cause of ALS both in vitro and in vivo. To our knowledge, this review is the first to focus on the functions of GSH in ALS conditions and its role as a potential therapeutic agent in ALS pathogenesis.

## 2. The Roles of GSH as an Antioxidant in the Nervous System

GSH is found at different levels in all cells. It accounts for approximately 95% of the total non-protein thiol groups in cells and is ubiquitously distributed in the body. High levels of GSH have been found in the nervous system [32]. Many studies have suggested that an intensive metabolic exchange of GSH occurs between astrocytes and neurons, whose interactions appear to be critical for neuronal GSH homeostasis and the protection of neurons in the brain against ROS and oxidative damage.

### GSH Synthesis and Cellular Distribution in Neurons

GSH is a tripeptide composed of glutamate, cysteine, and glycine. It is the most abundant thiol molecule found in tissues, including the brain, with a concentration of approximately 1–10 mM in the latter compared to approximately 2–3 mM in neurons, which is higher than that in the blood or cerebrospinal fluid (approximately 4 μM) [33,34,35,36]. GSH is synthesized in the cytosol by consecutive ATP-dependent reactions catalyzed by two enzymes, namely γ-glutamate-cysteine ligase (γ-GCL) and GSH synthetase (GS) [37,38]. γ-GCL mediates the first step of GSH synthesis, an ATP-dependent enzymatic process, with glutamate and cysteine to form the dipeptide, γ-glutamyl cysteine. γ-GCL is composed of a catalytic subunit (GCLC) and a modulatory subunit (GCLM), and is a rate-limiting enzyme in GSH synthesis [39]. Conditional *GCLC* knockout mice in whole neuronal cells displayed GSH depletion and neuronal cell death [40]. *GCLM* knockout mice exhibited reduced GSH levels and abnormal behavior phenotypes [41]. GS in the last step of GSH synthesis mediates the formation of GSH, which combines with glycine to form γ-glutamyl cysteine [42].

GSH is oxidized to glutathione disulfide (GSSG) by the reaction of glutathione peroxidase (GPx) coupled with the reduction of hydrogen oxide or hydroperoxides. GSH is regenerated from GSSG by GR, which uses NADPH as an electron donor [43]. Therefore, the reaction catalyzed by GPx and GR mediates GSH recycling. GSH is consumed by the extracellular release of GSH from cells via the generation of GSH-conjugates in the cytosol or oxidation to form GSSG. These processes induce a reduction in intracellular GSH. Thus, to maintain the intracellular levels of GSH, the synthesis, and recycling of GSH and the inhibition of GSH release must be induced. The depletion or redox imbalance of GSH (decreased GSH/GSSG ratio) has been reported to be involved in various neuronal dysfunction and neurodegenerative diseases, including autism, Alzheimer’s disease, Parkinson’s disease, and Huntington’s disease [44,45,46,47].

Although GSH is synthesized exclusively in the cytosol, it is present in the most important cellular organelles, namely the mitochondria, endoplasmic reticulum (ER), peroxisome, and nucleus [48]. These results suggest that GSH has specific functions in different cell compartments. Mitochondrial GSH represents 10–15% of the cellular GSH [49]. As the mitochondria lack the enzymes involved in de novo GSH synthesis, the maintenance of mitochondrial GSH levels depends on its uptake from the cytosol via carrier-mediated transporter systems. Many studies have shown that mitochondrial protection systems against free radicals and ROS, such as GSH, are critical for protecting neuronal cells from oxidative stress in the mitochondria. Moreover, mitochondrial GSH is important for protecting the organelles from ROS generated via the oxidative phosphorylation system. Muyderman et al. found that the selective depletion of mitochondrial GSH in astrocytes significantly increased hydrogen peroxide-induced apoptotic cell death and provided evidence for the crucial role played by mitochondrial GSH in preserving cell viability [50]. Wüllner et al. investigated the effects of acute GSH depletion and reduced mitochondrial GSH in relation to mitochondrial dysfunction in the cerebellar granule neurons of rats [51]. They found that the depletion of neuronal mitochondrial GSH led to a significant increase in ROS production and cell death in the nervous system. Furthermore, Wilkins et al. investigated the mechanism of mitochondrial GSH transport in the brain [52]. They showed that the dysfunction of mitochondrial GSH transporters, such as dicarboxylate and 2-oxoglutarate carriers, could result in an increased susceptibility of neurons to oxidative stress. These results demonstrate that the maintenance of mitochondrial GSH via sustained mitochondrial GSH transport is critical for protecting neurons from oxidative stress. Feng et al. also showed a reduction in mitochondrial GSH in the brain and spinal cord of *GCLC*-deficient mice [40]. Therefore, reduced mitochondrial GSH levels might be associated with mitochondrial defects in the nervous system.

The cytosol in cells is maintained in a reduced state to stabilize the free thiol groups. However, the ER environment is more oxidized than the cytosol to promote disulfide bond formation [48,53]. The redox environment in the ER influences the activity of various enzymes, including protein disulfide isomerase (PDI), which is responsible for the formation of disulfide bonds [53]. Previous studies have reported that the maintenance of redox status by regulating the GSH:GSSG ratio in the ER is considerably more oxidized than that in the cytosol [54]. Measuring the GSH levels in the ER revealed that the GSH:GSSG ratio in the ER is between 1:1 and 3:1 [55]. Furthermore, several studies have suggested the role of ER GSH in the formation of protein disulfide bonds; GSH is suggested to act as a net reductant in the ER by maintaining ER oxidoreductases, including PDI and endoplasmic reticulum oxidation 1 (ERO1) in a reduced state or by directly reducing non-native disulfide bonds in folding proteins [56,57,58]. Tsunoda et al. revealed that the selective depletion of ER GSH by expressing a cytosolic GSH-degrading enzyme, ChaC1, in the ER did not alter protein folding or ER stress response [59]. Although this study suggests the existence of an alternative electron donor that maintains the redox status in the ER, this result does not exclude the importance of the role of GSH in protein folding in the ER. However, direct evidence for the role of ER GSH in neuronal cells has yet to be found. However, as misfolded and aggregated proteins in the ER are one of the causes of neurodegenerative diseases, there is a need to determine the precise role of GSH in the ER of neurons.

GSH is present in the peroxisome—it is transported from the cytosol to the peroxisome by diffusion across the peroxisomal membrane [60,61]. Furthermore, GSSG is thought to be exported to the cytosol through a peroxisomal glutathione transporter, Opt2, and is subsequently reduced to GSH by cytosolic GR in an NADPH-dependent manner [62]. Catalase, peroxidase, and GSH are major components of the peroxisomal antioxidant system. GSH peroxidase (GPx) in the peroxisome requires GSH as a cellular reductant to reduce hydrogen peroxide to water [63].

GSH also plays a critical role in the nucleus. Previous studies have shown that the distribution of GSH to the nucleus is a critical factor in cell proliferation [64]. In plants, GSH depletion blocks the transition from G1 to S phase in the cell cycle of the root [65]. Moreover, GSH recruitment into the nucleus can regulate chromatin structure and condensation, which controls gene expression [66]. Therefore, the recruitment and translocation of GSH from the cytosol to the nucleus during the cell cycle has a great influence on cellular redox homeostasis and gene expression. Jeong et al. investigated GSH levels in living mammalian cells using a fluorescent real-time thiol tracer (FreSHtracer) [67]. They found that GSH levels were markedly higher in the nucleus than in the cytosol, and GSH was required for the maintenance of stem cell functions. Miller et al. also investigated the precise localization of GSH in the mouse central nervous system and found that GSH is synthesized in neurons and diffuses into the nucleus to protect DNA from oxidative stress [68]. However, the molecular functions of GSH in the nucleus of neurons and the mechanisms of GSH transport to the nucleus in neurons are not yet clearly understood and remain a topic of debate.

## 3. Evidence for the Dysfunction of GSH Metabolism in ALS

### 3.1. GSH Redox Imbalance in Cellular Models of ALS: In Vitro Studies

Extensive research has been conducted to investigate GSH metabolism and redox imbalance in experimental cellular models of ALS. As a result, an aberrant GSH redox balance, including GSH depletion in the nervous system, has been identified as a pathogenic mechanism of ALS in various in vitro models (Table 1). Lee et al. found that the levels of GSH and relative enzymes were decreased in familial ALS models [69]. They also examined the levels of GSH and GSSG in NT-2 cells (human teratocarcinoma cells) and SK-N-MC cells (human neuroblastoma cells) expressing human wild-type SOD1 or SOD1 mutants, including G37R and G85R, which are associated with fALS [69]. The GSH levels were found to be significantly reduced in NT-2 cells expressing SOD1^G37R^ or SOD1^G85R^. Furthermore, The GSH levels were significantly decreased in SK-N-MC cells expressing SOD1^G37R^ or SOD1^G85R^. However, the levels of GSSG in both mutants of the SOD1-expressing cells were increased. The activity of Gpx and protein carbonylation, a marker of protein damage induced by oxidative stress, was increased in cells transfected with the SOD1 mutants [69]. Rizzardini et al. showed that GSH depletion induced by ethacrynic acid impairs mitochondrial functions, including ROS production and membrane potential, in NSC-34 mouse motor neurons [70]. To investigate the effects of GSH depletion in motor neurons, NSC-34 cells were treated with ethacrynic acid, a GSH-depleting agent that directly conjugates GSH. The decreased GSH levels led to increased ROS, loss of mitochondrial membrane potential, and apoptosis. Furthermore, these researchers developed a cellular model of fALS by transfecting NSC-34 cells with human wild-type SOD1 or mutant SOD1^G94A^. SOD1^G94A^-expressing cells are more sensitive to mitochondrial dysfunction, including decreased mitochondrial membrane potential induced by ethacrynic acid, a GSH-depleting agent [71]. These results indicate that GSH depletion may be a suitable indicator in studies of the pathogenic mechanisms of oxidative stress-induced toxicity in motor neurons, suggesting that oxidative stress coupled with mitochondrial damage induces the development of disease onset similar to ALS.

Chi et al. investigated the effect of cellular GSH alterations on the cell death of motor neurons [72]. Their results showed that the treatment of NSC-34 cells with ethacrynic acid or L-buthionine sulfoximine significantly reduced GSH production and was accompanied by increased ROS generation. GSH depletion by ethacrynic acid enhanced the expression of the oxidative stress markers AP-1, c-Jun, c-Fos, and HO-1. Moreover, GSH depletion promoted apoptotic cell death by increasing cytochrome c release and capase-3 activation in NSC-34 cells [72]. Collectively, these results strongly suggest that decreased cellular GSH production and availability leads to increased intracellular oxidative stress and apoptosis, and promotes oxidative stress propagation and motor neuronal degeneration. Muyderman et al. also investigated the role of mitochondrial GSH in NSC-34 cells with human SOD1^G93A^. Interestingly, cells stably expressing SOD1^G93A^ showed significantly decreased mitochondrial GSH levels [73]. Furthermore, the treatment of SOD1^G93A^-expressing cells with ethacrynic acid resulted in increased apoptotic cell death and decreased the mitochondrial GSH pool. Such finding suggests that SOD1^G93A^ regulates mitochondrial oxidative stress by inducing the selective loss of the GSH pool in mitochondria.

Glutamate, an excitatory neurotransmitter in the mammalian nervous system, is a substrate for glutamate cysteine ligase together with cysteine in an ATP-dependent reaction [74]. This is the rate-limiting step in the synthesis of GSH, the main antioxidant in the central nervous system [74]. Abnormalities in both glutamate and glutamine levels were identified in the nervous tissues of a mouse model of fALS [75]. As a result, Cantoni’s group investigated the relationship between GSH synthesis and glutamine metabolism in a cellular model of fALS [76]. They found that both GSH and glutamate levels were significantly decreased in SOD1^G93A^-expressing NSC-34 cells cultured with the standard concentrations of glucose and glutamine, as well as alterations in the metabolic pathways involving glutamine/glutamate [76]. These results indicate that the decrease in GSH caused by SOD1^G93A^ expression is due to mitochondrial dysfunction associated with the reduction of the flux of glucose-derived pyruvate.

GSH production is regulated by the Nrf2 signaling pathway. This signaling pathway is impaired in various SOD1 models of fALS [77], and may therefore play a critical role in ALS pathogenesis. Moujalled et al. studied the GSH content in primary astrocytes from TDP-43^Q331K^-expressing mice and found a significant impairment in total GSH induction in response to sodium arsenite treatment in the astrocytes of TDP-43^Q331K^-expressing mice [78]. This result suggested that the mutant form of TDP-43 impairs the production of GSH by regulating the Nrf2 antioxidant signaling pathway. Moreover, Muyderman’s group showed that GSH is depleted, resulting in increased ROS levels in TDP-43^A315T^-expressing NSC-34 cells [79]. The depletion of GSH in TDP-43 ^A315T^-expressing cells or the loss of TDP-43 function resulted in increased intracellular ROS production, cell death, and cytosolic mislocalization of TDP-43, whereas protection against mutant TDP-43-mediated cytotoxicity was restored by increasing intracellular GSH levels by treatment with GSH monoethyl ester [79]. Collectively, these results suggest that oxidative stress is a critical factor in TDP-43-associated ALS pathogenesis and may result from the loss of GSH production. Therefore, novel therapeutics that restore GSH content in motor neurons may be beneficial in the prevention of TDP-induced neurotoxicity.

### 3.2. GSH Redox Imbalance in Animal Models of ALS: In Vivo Studies

**Figure 1:**
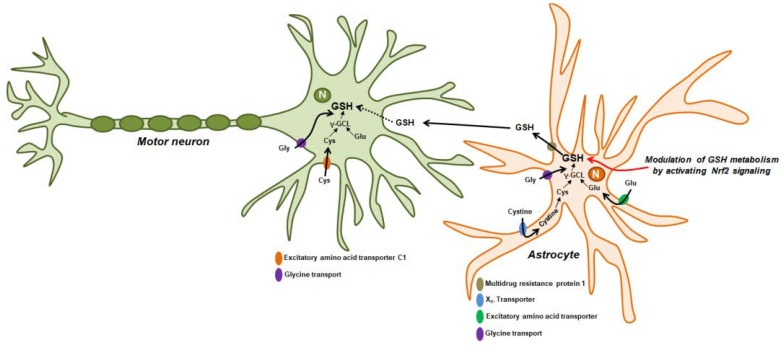
Astrocyte-mediated regulation of GSH biosynthesis and transport to motor neurons. The biosynthesis and release of GSH are associated with the Nrf2 signaling pathway in astrocytes [83]. Astrocytes take up cysteine, glutamate, and glycine through various transporters; thereafter, GSH is released from astrocytes via multidrug resistance protein 1 and transported to the motor neurons [104]. Cysteine and glutamate uptake are mediated by the X_c-_ transporter and excitatory amino acid transporter in astrocytes [91,105]. Glycine is transported by astrocytic glycine transport type 1 [106]. Intracellular GSH is synthetized from these amino acids in astrocyte [107]. Extracellular GSH may be taken up by motor neurons directly or indirectly [108]. Motor neurons also take up three amino acids for GSH synthesis. Increased GSH synthesis in astrocytes may exert a protective effect against motor neuronal toxicity under oxidative stress and in ALS.

Several in vitro studies have implicated GSH redox imbalance as a critical mediator of increased ROS generation and apoptosis in motor neurons and astrocytes. Mutations in ALS-causing genes have been found to lead to GSH depletion and neuronal toxicity. Therefore, there is a need to determine the precise role of GSH depletion and novel regulators of ALS pathogenesis. In particular, the novel regulatory mechanisms of GSH redox imbalance, including GSH depletion, dysfunction of GSH metabolism, and GSH transport, associated with ROS production in in vivo ALS models, need to be explored. Consistent with in vitro cellular studies, in vivo studies using various ALS animal models have provided evidence that the expression of ALS-causing genes activates various pathways and regulators that lead to a GSH redox imbalance. Astrocytes may play a critical role in the survival of motor neurons in ALS (Figure 1) [80]. According to previous studies, increased levels of GSH caused by activated astrocytes and released GSH from astrocytes improve the antioxidant status of co-cultured neurons [81,82,83,84]. Vargas et al. showed that GSH production in spinal cord astrocytes from SOD1^G93A^-expressing rats can prevent motor neuron apoptosis induced by nitric oxide [85]. Further, these researchers found that the neurotoxic effects of SOD1^G93A^ expression in astrocytes can be counteracted by increased GSH levels induced by the activation of the Nrf2 signaling pathway [85]. They also revealed that Nrf2 activation in astrocytes protects against neuronal toxicity in SOD1^G93A^ transgenic mice [86]. Nrf2 overexpression in astrocytes increased the survival rate and delayed neuromuscular denervation in SOD1^G93A^ transgenic mice. Furthermore, an increased GSH content was observed in the spinal cord and cerebellum tissues of SOD1^G93A^-coexpressing mice with Nrf2 [86]. Such a finding indicates that the activation of the Nrf2 signaling pathway can restore motor neuronal toxicity by increasing GSH synthesis and release in astrocytes (Figure 1).

Both Liu’s and Linseman’s groups studied the levels of GSH and GSSG in whole blood and the spinal cord lumbar region of SOD1^G93A^-expressing mice during disease progression [72,87]. As a result, decreased extracellular GSH levels were observed in the whole blood of SOD1^G93A^-expressing mice. Moreover, intracellular GSH levels in motor neurons were found to be significantly reduced during disease progression in the mice [87]. However, the GSSG levels were significantly increased in SOD1^G93A^-expressing mice [72]. Therefore, reduced GSH may contribute to motor neuron cell death by inducing the nuclear translocation of apoptosis-inducing factor (AIF) in SOD1^G93A^ transgenic mice. Vargas et al. examined the effect of decreased GSH in ALS models with the knockout of a modifier subunit of glutamate-cysteine ligase (GCLM) [88]. Decreased GSH was found to reduce survival and neurons in the ventral horn and spinal cord tissues of SOD1^G93A^-expressing mice with *GCLM* knockout. Moreover, increased ROS production and mitochondrial dysfunction were also observed in SOD1^G93A^-expressing mice with GCLM knockout [88]. The researchers also investigated the effects of *GCLM* loss in SOD1^WT^-expressing mice [89]. A reduction of approximately 70% in the total GSH content was observed in the brain cortex, brainstem, and spinal cord in the *GCLM* knockout of SOD1^WT^-expressing mice. Interestingly, the loss of *GCLM* resulted in a decreased survival, increased motor neuron loss, and accelerated muscle denervation in SOD1^WT^-expressing mice [89]. A reduction in GSH synthesis caused by the loss of *GCLM* may contribute to disease development and progression by modulating mitochondrial function in SOD1^WT^ and SOD1^G93A^-induced mouse models of ALS.

The cystine/glutamate antiporter (system X_C_-) is an important factor for GSH synthesis, tasked with transporting cystine into the cell to facilitate the release of glutamate into the extracellular space [90,91]. Cytoplasmic cystine is reduced to cysteine and is used in GSH synthesis in the brain [92,93]. Albano et al. revealed increased cystine uptake in the spinal cord of SOD1^G93A^ transgenic mice [94]. Therefore, it is possible that enhanced system X_C_- activity could be a protective mechanism for oxidative damage in motor neurons by maintaining intracellular GSH levels.

Treatment with urate has been found to suppress oxidative stress-induced toxicity in vitro [95]. Moreover, Serum uric acid levels were found to be reduced in patients with ALS [96,97]. Thus, decreased urate levels may be associated with the development and progression of neurodegenerative diseases, including ALS. Zhang et al. showed that treatment with urate increased the levels of GSH in the SOD1^G85R^-expressing *Drosophila* model of fALS by upregulating the Akt signaling pathway and catalytic subunit of glutamate-cysteine ligase (GCLC) [98]. This finding indicates that urate plays a neuroprotective role in motor neuronal damage by activating GSH synthesis.

Glucocorticoids are cholesterol-derived steroid hormones secreted exclusively by the adrenal gland and are critical regulators of homeostasis under basal and stress conditions [99,100]. Glucocorticoids are involved in excessive ROS production, which is associated with various neurodegenerative diseases, including TDP-43-induced proteinopathies [101,102]. Caccamo et al. found that dexamethasone, a synthetic glucocorticoid, increased the susceptibility to TDP-43-induced neurotoxicity in a mouse model [103]. Dexamethasone treatment was also found to exacerbate memory deficits and impair autophagy activation in the C-terminal fragment of TDP-43 and TDP-25-expressing mice. By examining the alteration of cellular redox status following dexamethasone treatment, the researchers found that the GSH/GSSG ratio was significantly decreased in dexamethasone-treated TDP-25-expressing mice [103]. The data indicate that there is a correlation between the decrease in the GSH/GSSG ratio and TDP-25-induced neurotoxicity in the brains after treatment with dexamethasone. Finally, accumulating evidence suggests that further research on the dysregulation of GSH metabolism and GSH-related enzymes induced by neurotoxic conditions could facilitate the development of a potential therapeutic strategy for ALS.

**Table 1:** Alteration of GSH content in various experimental models of ALS.

Cell Lines and Animals	Experimental ALS Models	Phenotypes Associated with Oxidative Stress	GSH Status	Reference
NT-2 SK-N-MC	Human SOD1^WT^ Human SOD1^G37R^ Human SOD1^G85R^	Increased protein carbonyl Increased 8-OHG Increased lipid peroxidation	Decrease GSH Increased GSSG	[69]
NSC-34	Ethacrynic acid treatment	Decreased mitochondrial membrane potential Increased ROS generation Increased apoptosis	Decreased GSH	[70]
NSC-34	Human SOD1^WT^ Human SOD1^G93A^	Decreased cell viability Increased ROS generation Decreased mitochondrial membrane potential Increased mitochondrial toxicity	-	[71]
NSC-34	Ethacrynic acid treatment	Increased ROS generation Increased oxidative response gene expression Increased apoptosis	Decreased GSH	[72]
NSC-34	Human SOD1^G93A^	Decreased cell proliferation Decreased cell viability Increased apoptosis	Decreased mitochondrial GSH	[73]
NSC-34	Human SOD1^WT^ Human SOD1^G93A^	Increased mitochondrial dysfunction	Decreased GSH	[76]
NSC-34	Human TDP-43^M337V^	Increased gene expression of Nrf2 signaling pathway	Decreased GSH	[78]
NSC-34	Human TDP-43^WT^ Human TDP-43^A315T^	Increased ROS generation Decreased cell viability Increased cell death	Decreased GSH	[79]
NSC-34	Human SOD1^G93A^	Restored cell viability by treating urate	Increased GSH by treating urate	[98]
Rat astrocytes	Human SOD1^G93A^	Restored cell survival by activating Nrf2	Increased GSH by activating Nrf2	[85]
Mouse motor neurons	Human SOD1^G93A^	Increased apoptosis	Decrease GSH Increased GSSG	[72]
Mouse astrocytes	Human SOD1^G93A^	Extended survival by expressing Nrf2 Delayed muscle denervation by expressing Nrf2	Increased GSH secretion by expressing Nrf2	[86]
Mouse astrocytes	Human SOD1^G93A^ Human SOD1^H46R/H48Q^	Decreased cell survival by *GCLM* knockout Increased motor neuron loss by *GCLM* knockout Increased oxidative stress by *GCLM* knockout Decreased complex IV activity by *GCLM* knockout	Decrease GSH by *GCLM* knockout	[88]
Mouse	Human SOD1^G93A^	Increased cystine uptake by cystine/glutamate antiporter	-	[94]
Fly	Human SOD1^WT^ Human SOD1^G85R^	Extended survival by treatment with urate Improved motor defect by treatment with urate Enhanced antioxidant enzyme activity by treatment with urate Decreased ROS level by treatment with urate	-	[98]
Mouse	Human TDP-25	Increased memory deficit by treatment with dexamethasone	Decreased GSH/GSSG ratio by treatment with dexamethasone	[103]
Mouse	Human SOD1^G93A^	Decreased survival	Decreased GSH in whole blood and spinal cord	[87]
Mouse	Human SOD1^WT^	Decreased cell survival by *GCLM* knockout Increased motor neuron loss by *GCLM* knockout	Decrease GSH by *GCLM* knockout	[89]

### 3.3. GSH Redox Imbalance in ALS Patients

Previous studies have identified binding sites for GSH in the synaptic membranes of the brain and spinal cord [109,110]. In 1993, Lanius et al. examined GSH binding in the spinal cord of sALS patients using a radioactively labeled GSH, [^35^S]-GSH, and found that [^35^S]-GSH binding in the spinal cord was increased in patients with sALS [111]. Similarly, Babu et al. observed an imbalance in the antioxidant system in the erythrocytes of patients with sALS [28]. In fact, they found that GSH levels were significantly decreased in the erythrocytes of patients with sALS. Intracellular GSH recycling is catalyzed by GR using NADPH as a reducing agent to convert GSSG to two molecules of GSH [112]. GR activity was also found to be reduced in sALS patients compared to that in control patients [28]. Another group found that erythrocyte GPx activity was significantly impaired in patients with sALS, which remained low during disease progression [29]. GPx catalyzes the detoxification of hydrogen peroxide by utilizing GSH as a major source of protection against oxidative damage in the nervous system [113]. Thus, it can be hypothesized that an antioxidant imbalance, including the depletion of GSH and GSH-related enzymes, could be a contributing factor to the development and progression of ALS.

Weiduschat et al. investigated the in vivo levels of GSH in the motor cortex region of patients with ALS using the J-edited spin-echo difference magnetic resonance spectroscopy (MRS) technique [114]. Consistent with this study, other groups have reported a decrease in GSH levels in the motor cortex and corticospinal tract in patients with ALS compared to healthy controls [115,116]. The GSH levels in the corticospinal tract were more strongly correlated with disease progression than those in the motor cortex. Decreased GSH levels in the motor cortex of patients with ALS are likely to be a manifestation of clinical changes and pathogenic processes specific to ALS. Yang et al. also performed a Mendelian randomization analysis to identify ALS-associated metabolites, and detected 18 metabolites, including γ-glutamyl amino acids, that may exert causal effects on the development and progression of ALS [117]. The γ-glutamyl cycle is responsible for GSH biosynthesis [118]. Therefore, oxidative damage caused by the dysfunction of GSH metabolism may be a critical risk factor associated with the pathogenesis of ALS.

## 4. Clinical Trials in ALS

In 1998, Schiffer’s group studied the effect of GSH treatment on the rate of progression of ALS in patients and found that GSH treatment did not have a significant effect on ALS progression [119]. Some cysteine-containing molecules, such as N-acetylcysteine (NAC) and procysteine, are used to increase GSH levels. Brown’s group studied the pharmacokinetic properties of procysteine, a cysteine prodrug that increases intracellular GSH levels in patients with ALS [120]. However, the subcutaneous infusion of NAC did not reduce disease progression in patients with ALS [121]. Of note, the GSH levels in the cerebrospinal fluid of patients with ALS are markedly reduced with aging. Although the oral administration of cysteine-containing molecules did not have statistically beneficial effects on ALS patients, cysteine-containing molecules may be a valuable therapeutic option given its ability to increase GSH content in the cerebrospinal fluid.

## 5. Conclusions

GSH exerts important functions in the central nervous system as an antioxidant, enzyme cofactor, redox buffer, and neuromodulator. GSH deficiency and the dysfunction of GSH metabolism are common to several neurodegenerative diseases, including Alzheimer’s disease and Parkinson’s disease. Although accumulating evidence suggests that aberrant GSH homeostasis is linked to the development and progression of ALS, the precise role and mechanism of redox imbalance in neuronal cells in this disease remains to be determined. In this review, we first summarized the experimental evidence supporting the role of GSH in the development and progression of ALS. Many studies on ALS have focused on the decreased GSH content and imbalanced GSH redox status in the nervous system, which result in ALS progression. Furthermore, several GSH-related enzymes that target oxidative stress and regulate GSH homeostasis have also been studied. The mechanism of ALS progression may involve decreased GSH, increased GSSG, and an impaired GSH-associated antioxidant system, suggesting that an imbalance in redox status beyond its physiological limit may be detrimental to motor neuronal functions and survival that regulate disease initiation and conditions. Although several studies provide indirect or direct evidence that the dysregulation of GSH homeostasis and metabolism in the nervous system are associated with the pathogenesis of ALS, it remains unclear whether reduced GSH is a causative factor in ALS or whether various mutations in ALS-causing genes are responsible for the impaired GSH redox status observed in ALS. In addition, the molecular mechanisms and pathways related to the synthesis, transport, and degradation of GSH in neurons and astrocytes are complex, and our understanding of the imbalanced GSH redox status in the brain of ALS patients remains incomplete. Therefore, future studies should aim to intensively investigate whether treatments that increase the GSH/GSSG ratio in motor neurons and astrocytes by activating GSH biosynthesis have clinical efficacy in the treatment of ALS. In addition, there is a need to study and develop effective pharmacological agents that may enhance GSH functions to reduce oxidative stress and damage cellular organelles, including the mitochondria, in motor neurons.
